# Condensin I Recruitment to Base Damage-Enriched DNA Lesions Is Modulated by PARP1

**DOI:** 10.1371/journal.pone.0023548

**Published:** 2011-08-12

**Authors:** Xiangduo Kong, Jared Stephens, Alexander R. Ball, Jason T. Heale, Daniel A. Newkirk, Michael W. Berns, Kyoko Yokomori

**Affiliations:** 1 Department of Biological Chemistry, School of Medicine, University of California Irvine, Irvine, California, United States of America; 2 Department of Developmental and Cell Biology, Beckman Laser Institute, University of California Irvine, Irvine, California, United States of America; University Medical Center Hamburg-Eppendorf, Germany

## Abstract

Condensin I is important for chromosome organization and segregation in mitosis. We previously showed that condensin I also interacts with PARP1 in response to DNA damage and plays a role in single-strand break repair. However, whether condensin I physically associates with DNA damage sites and how PARP1 may contribute to this process were unclear. We found that condensin I is preferentially recruited to DNA damage sites enriched for base damage. This process is dictated by PARP1 through its interaction with the chromosome-targeting domain of the hCAP-D2 subunit of condensin I.

## Introduction

Base excision repair (BER) is the primary cellular mechanism to address DNA base damage, which results from both endogenous and exogenous agents such as reactive oxygen species, alkylating agents, and ionizing irradiation [1]. Base damage is processed by DNA glycosylases and AP endonuclease into a single-strand break (SSB) intermediate that is then further repaired. Poly(ADP-ribose) polymerase 1 (PARP1) acts as a DNA nick-sensor that is thought to organize the damage site chromatin and/or serve as a scaffold together with its binding partner XRCC1 for subsequent recruitment of repair proteins in BER as well as SSB and double-strand break (DSB) repair [2], [3], [4]. The catalytic activity of PARP1 is activated in the presence of DNA damage, which leads to ADP-ribosylation of itself and of its target proteins. Auto-ADP-ribosylation of PARP1 results in its dissociation from chromatin. Although both linker and core histones are well-described substrates of PARP1, the identities of the key target proteins at the damage sites have not been completely established. Nonetheless, poly(ADP-ribose) (PAR) enriched at the damage sites was recently shown to serve as an important binding platform for several DNA repair and chromatin-modifying factors, indicating that PARP1 plays an important role in local chromatin organization at the damage sites [5], [6], [7], [8], [9].

Condensins are essential for normal mitotic chromosome organization and segregation [10]. There are two condensin homologs in higher eukaryotes, condensin I and condensin II, which share the same SMC heterodimer (hCAP-C-hCAP-E) but have different non-SMC subunits [11]. Each affects the organization and resolution of mitotic chromosomes in distinct ways, although the underlying mechanisms are not well understood [11]. In human cells, condensin I contains three unique non-SMC subunits termed hCAP-D2 (CNAP1/Eg7), hCAP-G, and hCAP-H [12], [13]. We previously reported that human condensin I interacts directly with PARP1 in a DNA damage-induced manner, and plays a role in BER/SSB repair [14]. DNA damage increases chromatin association of condensin I together with PARP1 and XRCC1. However, how condensin I contributes to DNA repair, and how PARP1 impacts its function, are unresolved. Here, we report that condensin I is recruited to DNA damage sites enriched for base damage, revealing its direct role in the DNA damage response and its preference for a specific type of damage. We found that the same domain active in mitotic chromosome association also plays a critical role in damage site association by interacting with PARP1. Our results reveal the direct, yet PARP1 modulated, involvement of condensin I in mammalian base damage/SSB repair.

## Results

### Condensin I accumulates at base damage sites

**Figure 1:**
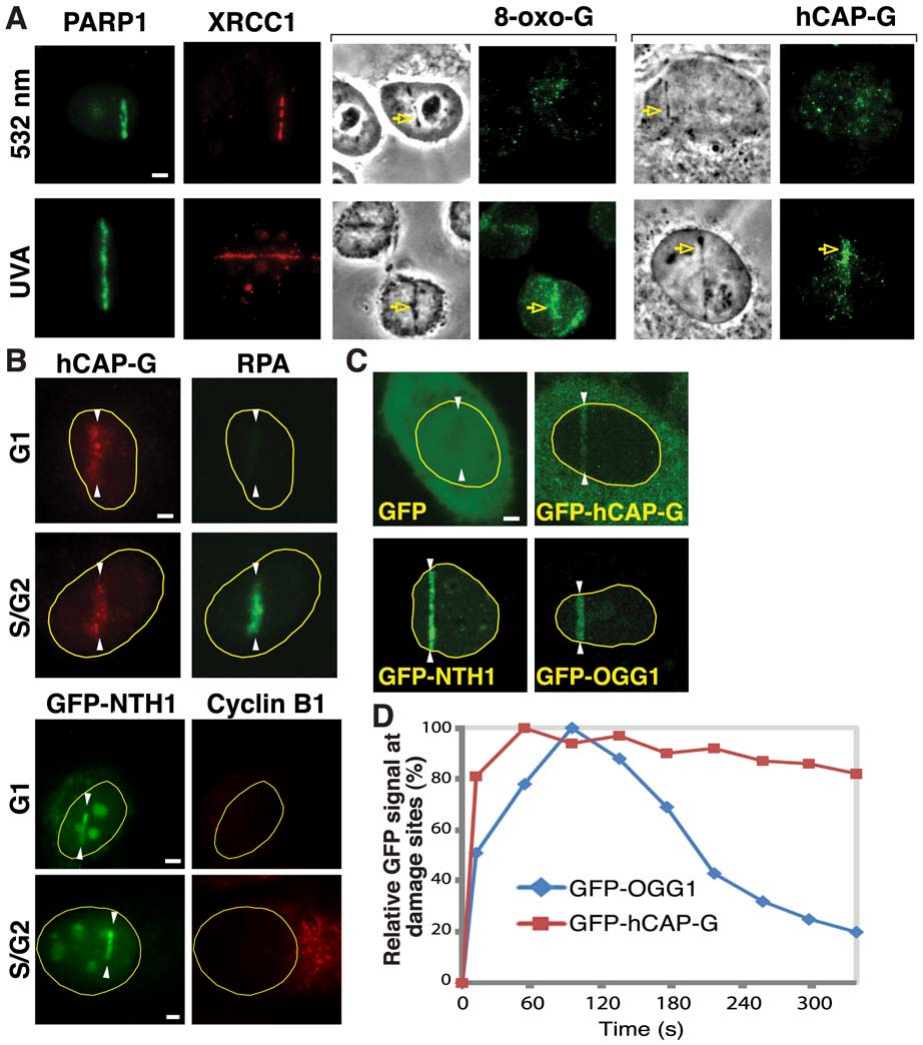
Condensin I is recruited to DNA damage sites. (**A**) Antibodies specific for 8-oxoG (marker for base damage), PARP1, XRCC1, and the condensin I subunit hCAP-G were used to stain cells damaged by green (532 nm) or UVA lasers as indicated. Scale bar = 5 µm for the rest of the study. The damage sites are indicated by arrows. (**B**) Cells in G1 or S/G2 phase were damaged using the NIR laser and the localization of the endogenous hCAP-G was detected by immunofluorescent staining. Cells were costained with antibody specific for RPA, which served as a marker for S/G2 phase. Similarly, GFP-NTH1 (see (C)) localization at NIR-induced damage sites was examined in G1 and S/G2 phase. Cyclin B1 staining was used as a marker for S/G2. White arrowheads indicate the damage sites. (**C**) Stable cell lines expressing either GFP-hCAP-G or GFP alone, and cells transiently expressing GFP-NTH1 or GFP-OGG1, were cut with the NIR laser and GFP clustering to the damage sites was examined live at two min after damage induction. White arrowheads indicate the damage sites. (**D**) Cells expressing GFP-hCAP-G or GFP-OGG1 were damaged and accumulation of the GFP signal at the damage sites was measured over time in live cells. Relative GFP signals (%) were calculated using the peak accumulation of the GFP signal at the damage sites in each cell line as 100%.

Although overall chromatin association of condensin I is increased in response to DNA damage [14], it was unclear whether condensin I actually localizes to the damage sites. We used laser microirradiation to detect potential recruitment of condensin I to laser-induced damage sites. We previously found that different laser conditions result in different amounts of base damage [15]. The nanosecond (ns) UVA (337 nm) laser, but not, for example, the ns green (532 nm) laser, induces significant base damage accompanied by robust recruitment of DNA glycosylases [15]. Using these two lasers, we found that hCAP-G, a non-SMC subunit of condensin I, is recruited to UVA-induced damage sites but not to green laser-induced damage sites by immunofluorescent staining (Fig. 1A). In contrast, PARP1 and XRCC1 recruitment was observed at both damage sites, reflecting their involvement in the repair of SSBs and DSBs generated by both lasers (Fig. 1A) [15]. Although the UVA laser induces strand breaks in addition to base damage [15], efficient recruitment of DNA glycosylases suggests that initial base damage is converted to SSBs, which also attract PARP1. In addition, condensin I is recruited to near-infrared (NIR) laser (780 nm) damage under conditions that also induced the efficient recruitment of green fluorescent protein (GFP)-tagged DNA glycosylases NTH1 and OGG1 [16] (Fig. 1B,C). Consistent with the fact that BER is active in both the G1 and S/G2 phases of the cell cycle, damage site recruitment of condensin I and GFP-NTH1 was observed in both G1 and S/G2 (Fig. 1B). Detection of other condensin I subunits at the lesion supports the presence of the condensin I holo-complex at the damage sites (Fig. S1). Thus, the results indicate that condensin I is indeed targeted to the damage sites, suggesting its direct role in DNA repair. Furthermore, the recruitment of condensin I to UVA- and NIR-induced damage sites bearing substantial base damage, but not to the green laser lesions containing strand breaks but no detectable base damage, suggests that condensin I preferentially recognizes base damage or base damage-derived SSBs.

In order to substantiate the antibody staining results, we generated a HeLa cell line that stably expresses GFP-tagged hCAP-G. GFP-hCAP-G is incorporated into the condensin I complex and exhibits proper subcellular localization during the cell cycle (Fig. S2). We found that GFP-hCAP-G, but not GFP alone, is recruited to the laser-induced damage sites similar to the endogenous protein (Fig. 1C). GFP-hCAP-G was recruited rapidly, reaching a peak within 60 seconds (sec) after damage induction (Fig. 1D). This is faster than the accumulation of GFP-OGG1. GFP-OGG1 rapidly disappeared from the damage sites with a half-life of less than 200 sec, consistent with the fact that DNA glycosylases act at an early stage of base damage repair [3], [17]. In contrast, a significant amount of GFP-hCAP-G remained at the damage sites for at least 300 sec, suggesting that condensin I may be involved in both early and late steps of BER.

### PARP1 affects condensin I binding to damage sites

**Figure 2:**
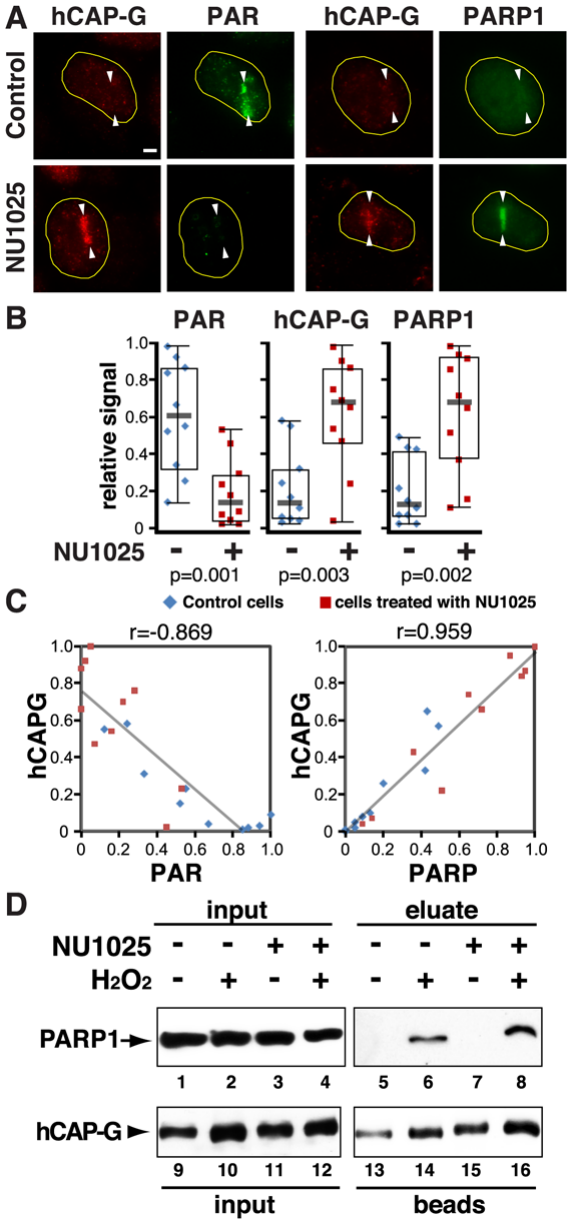
PARP1 inhibition enhances and prolongs PARP1 and condensin I accrual at the damage sites, and does not impair the condensin I-PARP1 interaction. (**A**) Cells were damaged in the presence or absence of NU1025 (100 µM) and fixed at one hour after irradiation. Cells were stained with antibodies specific for PAR, hCAP-G, and PARP1 as indicated. (**B**) Comparison of the relative signals of each antibody staining in the control DMSO-treated and NU1025-treated cells as in (A). Ten cells were measured in each group. The relative signal was calculated based on the highest value of fluorescent signal in each antibody group and displayed as boxplots with medians. The p values were generated using a t-test and are shown at the bottom. (**C**) Correlation of the hCAP-G/PAR and hCAP-G/PARP1 co-staining in individual cells is plotted with trend lines. Pearson's r values are shown at the top. (**D**) Co-IP of PARP1 with condensin I from HeLa nuclear extracts using anti-hCAP-G antibody with or without NU1025 in the presence or absence of damage.

Based on the above results, we tested the possibility that PARP1 may affect damage site binding of condensin I in vivo. We first tested whether the inhibition of PARP1 enzymatic activity has any effect on condensin I localization to lesions. Although PARP1 is rapidly recruited to damage sites, it dissociates in less than two hours [18]. At one hour after damage induction, while the PAR signal persists, the majority of PARP1 has dissociated (Fig. 2A, control). Treatment of cells with the PARP inhibitor NU1025 strongly inhibited the PAR signal at the damage sites (Fig. 2A,B). Since ADP-ribosylation of PARP1 results in its dissociation from chromatin, inhibiting PARP1 enzymatic activity significantly enhanced PARP1 retention at the damage sites (Fig. 2A,B). We demonstrated previously that condensin I is induced to interact specifically with the hypo-ADP-ribosylated form of PARP1, which is capable of binding to DNA, in response to DNA damage [14]. Consistent with this, we observed that endogenous hCAP-G localization at the damage sites also increased significantly in the presence of NU1025 (Fig. 2A,B). Similar results were obtained with a second PARP inhibitor (3-aminobenzamide (3AB)) (data not shown). Analysis of individual cells revealed that there is an inverse correlation between hCAP-G recruitment and PAR, and a direct correlation between hCAP-G and PARP1 recruitment either in the presence or absence of NU1025 (Fig. 2C). Thus, inhibition of PARP activity and/or enhancement of PARP1 retention at the damage sites potentiates condensin I accumulation at the damage sites. We confirmed that the damage-induced interaction between condensin I and PARP1 is not affected by NU1025 treatment, suggesting that the interaction is PAR-independent (Fig. 2D). Similar results were obtained using 3AB (data not shown).

**Figure 3:**
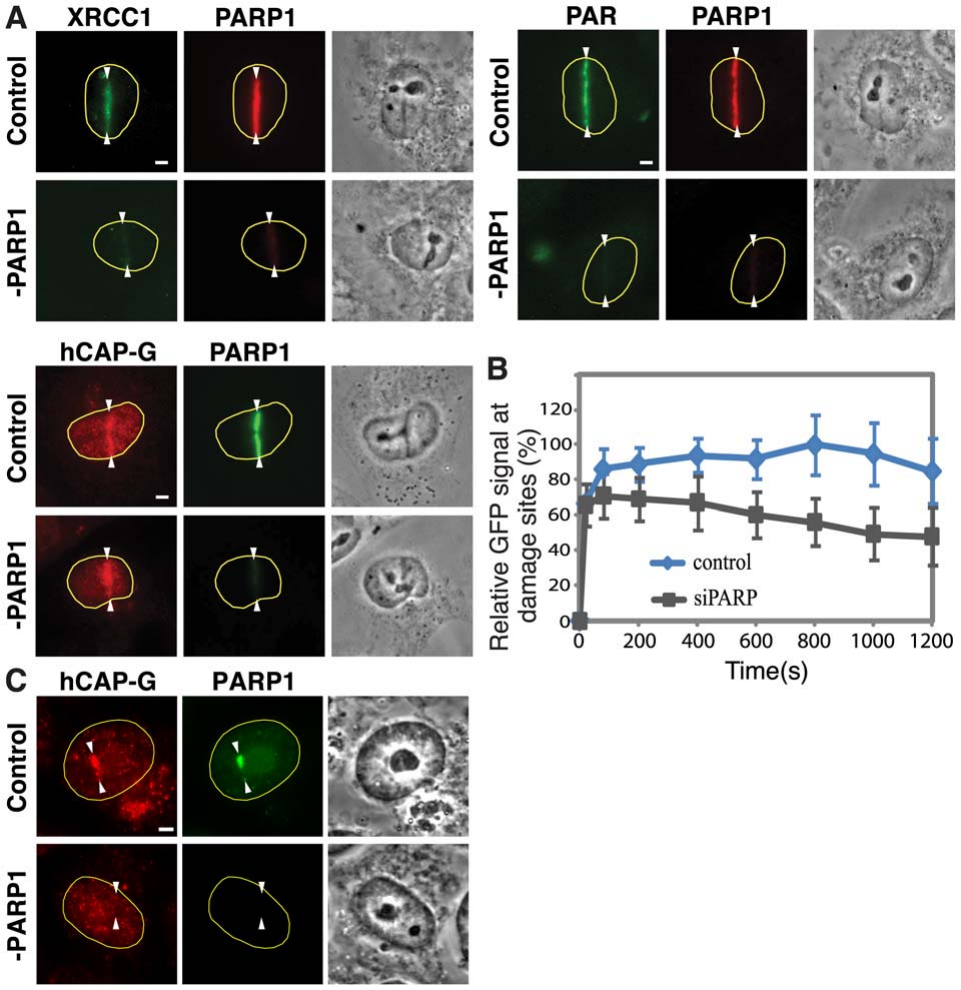
PARP1 depletion does not impair the initial recruitment of condensin I but affects its retention at the damage sites. (**A**) Cells were treated with siRNA specific for PARP1 or control siRNA, and stained with antibodies specific for PAR, XRCC1, PARP1, or hCAP-G at five min after damage induction. (**B**) Cells were treated with PARP1 or control siRNA as in (A) and the accumulation of GFP-hCAP-G was plotted over time as in Fig. 1D. (**C**) Cells treated with NU1025 were also treated with PARP1 or control siRNA and the accumulation of the endogenous hCAP-G and PARP1 were examined at one hour after damage induction.

In order to determine whether the PARP1 protein itself is required for condensin I localization to the damage sites, PARP1 was depleted by siRNA. PARP1 depletion abolished the PAR signal and the recruitment of XRCC1 to the damage sites (Fig. 3A). Condensin I, however, was detected at the damage sites five minutes after damage induction under these conditions. The results indicate that PARP1 is not required for the initial recruitment of condensin I to lesions. Consistent with this, the damage-induced chromatin association of condensin I is not affected in PARP1 knockout cells (Fig. S3). Thus, condensin I and PARP1 recognize damage independently of each other.

Interestingly, however, further kinetic analysis of GFP-hCAP-G accumulation at the damage sites with and without PARP1 siRNA treatment revealed that while the initial recruitment of GFP-hCAP-G to the damage sites is not affected by PARP1 depletion, its subsequent retention was compromised (Fig. 3B). Furthermore, the prolonged condensin I localization at damage sites at one hour post-irradiation in NU1025-treated cells (as in Fig. 2A) was diminished by PARP1 siRNA depletion (Fig. 3C). Taken together, the results indicate that PARP1 stabilizes condensin I association with DNA damage independent of its ADP-ribosylase activity.

### hCAP-D2 NCTD in PARP1-dependent damage response

**Figure 4:**
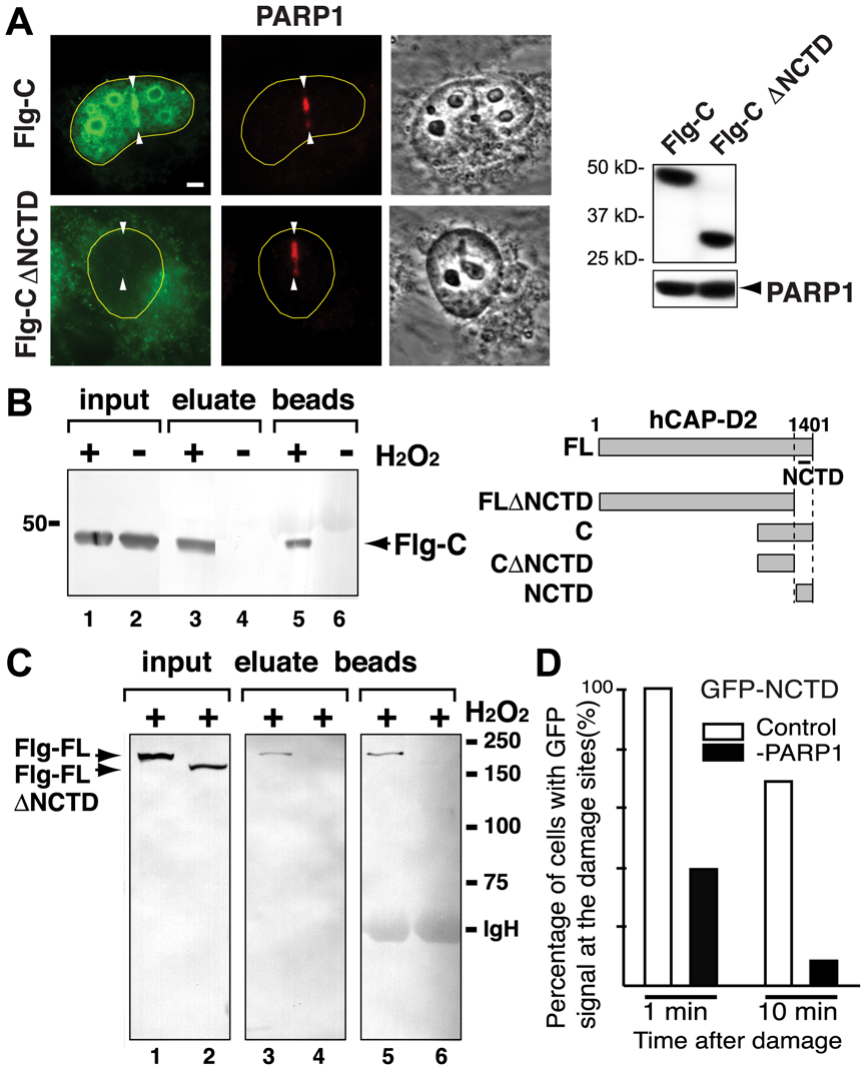
The hCAP-D2 NCTD is important for damage site targeting and damage-induced PARP1 interaction. (**A**) The recruitment of the FLAG (Flg)-hCAP-D2 C-terminal 400 a.a. region (Flg-C) and Flg-CΔNCTD to damage sites was examined by immunofluorescent staining using anti-FLAG antibody. PARP1 staining was used as a control. Comparable expression of Flg-C and Flg-CΔNCTD was confirmed by western analysis using anti-FLAG antibody. PARP1 was used as a loading control shown underneath. (**B**) HeLa cells expressing Flg-C were treated with or without H_2_O_2_ and the nuclear extracts were immunoprecipitated with anti-PARP1 antibody. FLAG-tagged proteins were detected by Western analysis using anti-FLAG antibody. Schematic diagrams of the recombinant proteins are shown. (**C**) Co-IP of FLAG-tagged full-length hCAP-D2 (Flg-FL), and the full-length without the NCTD region (Flg-FLΔNCTD), with anti-PARP1 antibody following H_2_O_2_ treatment. IgG heavy chain (IgH) is indicated. (**D**) Laser damage was introduced in control siRNA- and PARP1 siRNA-treated cells (30 cells each) and the recruitment of GFP-NCTD was examined.

We previously reported that the carboxy (C)-terminal 113 amino acid segment of hCAP-D2 contains a domain, termed the nuclear- and chromosome-targeting domain (NCTD), that has mitotic chromosome targeting activity and interacts directly with histones H1 and H3 [19]. Interestingly, the NCTD also directly interacts with PARP1 [14]. To further address the molecular mechanism of DNA damage recognition by condensin I and the stabilizing role of PARP1 in this process, we tested whether the hCAP-D2 NCTD is also important for DNA damage site recruitment. We fused the C-terminal 400 residues of hCAP-D2 (which includes the NCTD) to either FLAG or GFP, and found that this segment is sufficient for damage site localization. Deleting the NCTD from this fusion construct abolished its damage site targeting activity (Fig. 4A). The results indicate that the NCTD, important for mitotic chromosome binding, is also involved in damage site association.

We found that the same C-terminal region of hCAP-D2 is induced to interact with PARP1 in response to damage, and the damage-induced interaction of the full-length hCAP-D2 with PARP1 is disrupted when the NCTD is deleted (Fig. 4B,C). PARP1 depletion significantly compromised the damage site targeting of GFP-hCAP-D2 NCTD, indicating that the damage targeting activity of this domain requires PARP1 (Fig. 4D). Thus, the results suggest that PARP1 modulates condensin I association with damaged chromatin through the hCAP-D2 NCTD.

## Discussion

Although condensin I binds to chromosomes in mitosis, the majority of condensin I resides in the cytoplasm during interphase with a small population remaining in the nucleus [13], [14]. How this residual nuclear localization of condensin I is regulated is currently unclear. Nevertheless, our results show that this nuclear condensin I is recruited to DNA damage sites together with PARP1.

We previously demonstrated that cohesin, another major SMC-containing complex that functions in sister chromatid cohesion [20], is recruited to green laser-induced damage sites in a S/G2-specific manner [21], consistent with its role in post-replicative DSB repair [22], [23], [24]. Condensin I, however, failed to localize to green laser-induced damage sites that contain strand breaks but not base damage [15], revealing the specificity of damage recognition by the complex. These functional specificities appear to be linked to distinct protein interactions. Cohesin interacts with and requires the Mre11 complex for its damage site targeting [21]. Condensin I interacts with PARP1, XRCC1, and several other BER factors, but not the Mre11 complex [14].

Interestingly, the hCAP-D2 NCTD functions in both mitotic chromosome binding and damage site targeting, but only the latter requires PARP1, suggesting that PARP1 is critical for damage-specific NCTD regulation [14]. However, condensin I does not localize to the green laser-induced damage sites that efficiently recruit PARP1, and the initial recruitment of condensin I is PARP-independent. This suggests the presence of an additional domain(s) in condensin I that dictates damage recognition specificity.

It is currently unclear why condensin I, which functions in chromosome condensation in mitosis, is recruited to the damage sites. Although this seems counterintuitive, PARP1 was recently shown to recruit heterochromatin factors (i.e., macroH2A, polycomb and NuRD complexes) to damage sites, raising the intriguing possibility that chromatin may not simply become “relaxed and open” for DNA repair [5], [6], [7], [25]. Interestingly, these factors are recruited to DNA damage sites by directly binding to PAR and affecting DSB repair. Condensin I, however, which is important for BER and SSB (but not DSB) repair, is stabilized at the damage sites by direct interaction with the PARP1 protein. Thus, the results reveal different modes of chromatin regulation at damage sites by PARP1. Our results provide important evidence for the direct involvement of condensin I, together with PARP1, in the base damage response at damage sites, providing an important basis to further explore chromatin regulation in DNA repair.

## Methods

### Cell culture and plasmid constructs

HeLa cells (ATCC) were grown as described [19]. The tagged hCAP-D2 full-length and deletion mutants were described previously [19]. GFP-NTH1 and GFP-OGG1 expression constructs were transfected using PolyFect (Qiagen). At 20 hours after transfection, cells were damaged and their clustering was examined. GFP alone or GFP-hCAP-G in pIRESneo3 (BD Biosciences Clontech) was used to generate stable HeLa cell lines.

### Antibodies

Antibodies specific for hCAP-G, PARP1 and hCAP-H were previously described [14]. Mouse monoclonal antibodies specific for PARP1 and PAR polymers (Biomol Research Laboratories Inc.), XRCC1 (Gene Tex, Inc.), 8-oxoguanine (8-oxo-G, Trevigen, Inc), RPA (Millipore), and FLAG (Sigma), and rabbit polyclonal antibody specific for Cyclin B1 (Cell Signaling) were also used.

### Co-immunoprecipitations (co-IPs)

Nuclear extract preparation and co-IPs were done as previously described [14], [19]. DNA damage for the co-IP experiments was induced by treatment of cells with 20 mM H_2_O_2_ for 20 min at 37°C as described [14]. Immunoprecipitated materials on beads were washed first with low salt followed by 1 M salt, and then eluted with 2 M guanidine-HCl. Eluate fractions and beads were analyzed by western blotting.

### Laser systems and image analyses

DNA damage by a 532 nm green laser or a UVA laser was performed as previously described [15]. DNA damage by NIR femtosecond (fs) laser irradiation was carried out using a Zeiss LSM 510 META multiphoton-equipped (3.0W 170 fs Coherent tunable Chameleon Ultra NIR laser) confocal microscope. The chameleon NIR beam was tuned to the 3 photon absorption peak of DNA (780 nm) where the software bleach function was used to target linear tracts inside the cell nuclei for exposure to single laser scans (6.3 µs pixel dwell time, 1.33–1.59×10^12^ W/cm^2^) through the 100X objective (1.4 NA Zeiss Plan APO). Recruitment of GFP-tagged proteins to damage sites was observed by live-cell confocal scanning with the 488 nm CW argon laser on the same Zeiss META platform. Endogenous proteins were detected by fixation and immunostaining as previously described [15]. Fluorescent images were captured through a 100× Ph3 UPlanFI oil objective (NA 1.3; Olympus) on a Model IX81 Olympus microscope with a CCD camera. The immunofluorescent signals at damage sites were measured with MicroSuite™ FIVE Imaging Software (Olympus). The experiments were repeated at least three times and each time six to seven cells were examined, which showed consistent results.

### siRNA transfection

A mixture of two siRNAs against PARP1 (5′-CCG AGA AAT CTC TTA CCT CAA-3′ and 5′-ACG GTG ATC GGT AGC AAC AAA-3′) and the control siRNA (QIAGEN) were transfected into HeLa cells twice at a 24 hr interval using HiPerFect following the manufacturer's instructions (QIAGEN).

## Supporting Information

Figure S1 Immunofluorescent detection of condensin I subunits at the laser-induced damage sites. Antibodies specific for the non-SMC subunit hCAP-D2 and the SMC subunit hCAP-E were used to detect the endogenous proteins at the damage sites.
(PDF)

Figure S2 (A) Live fluorescent images of GFP-hCAP-G localizing to the cytoplasm in interphase and chromosomes in mitosis. This is in contrast to GFP alone, which distributes evenly in both the cytoplasm and the nucleus in interphase and is excluded from chromosomes in mitosis. Live Hoechst 33342 was used to visualize DNA. Scale bars = 5 µm. (B) Incorporation of GFP-hCAP-G into the condensin I complex was confirmed by co-IP with anti-GFP antibody and the precipitates were probed with a mixture of antibodies against hCAP-C, hCAP-E, hCAP-D2, and hCAP-H. The co-IP pattern was compared to that of the untransfected HeLa extracts using anti-hCAP-G antibody.
(PDF)

Figure S3 Chromatin association of condensin I in PARP1 knockout cells. The wild type and PARP1 knockout mouse embryonic fibroblasts (MEFs) [26] were treated with or without H_2_O_2_ and chromatin fractions were purified by CSK extraction [14] and probed for mouse CAP-D2 and PARP1 by western blotting.
(PDF)
