# Supplementary material for: Condensin I Recruitment to Base Damage-Enriched DNA Lesions Is Modulated by PARP1
Source: PLoS One. 2011 Aug 12;6(8):e23548. doi: 10.1371/journal.pone.0023548 (PMC3155556; doi:10.1371/journal.pone.0023548)

Figure S1. Immunofluorescent detection of condensin I subunits at the laser-induced damage sites. Antibodies specific for the non-SMC subunit hCAP-D2 and the SMC subunit hCAP-E were used to detect the endogenous proteins at the damage sites.

Figure S1

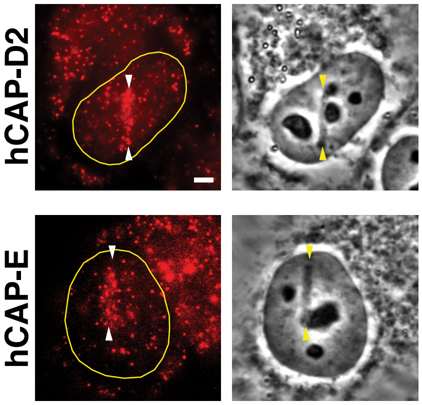

Supplement: Figure S1 — Immunofluorescent detection of condensin I subunits at the laser-induced damage sites. Antibodies specific for the non-SMC subunit hCAP-D2 and the SMC subunit hCAP-E were used to detect the endogenous proteins at the damage sites. (PDF) [file pone.0023548.s001.pdf]
