# Supplementary material for: Condensin I Recruitment to Base Damage-Enriched DNA Lesions Is Modulated by PARP1
Source: PLoS One. 2011 Aug 12;6(8):e23548. doi: 10.1371/journal.pone.0023548 (PMC3155556; doi:10.1371/journal.pone.0023548)
Supplement: Figure S2 — (A) Live fluorescent images of GFP-hCAP-G localizing to the cytoplasm in interphase and chromosomes in mitosis. This is in contrast to GFP alone, which distributes evenly in both the cytoplasm and the nucleus in interphase and is excluded from chromosomes in mitosis. Live Hoechst 33342 was used to visualize DNA. Scale bars = 5 µm. (B) Incorporation of GFP-hCAP-G into the condensin I complex was confirmed by co-IP with anti-GFP antibody and the precipitates were probed with a mixture of antibodies against hCAP-C, hCAP-E, hCAP-D2, and hCAP-H. The co-IP pattern was compared to that of the untransfected HeLa extracts using anti-hCAP-G antibody. (PDF) [file pone.0023548.s002.pdf]

Figure S2. (A) Live fluorescent images of GFP-hCAP-G localizing to the cytoplasm in interphase and chromosomes in mitosis. This is in contrast to GFP alone, which distributes evenly in both the cytoplasm and the nucleus in interphase and is excluded from chromosomes in mitosis. Live Hoechst 33342 was used to visualize DNA. Scale bars=5μm. (B) Incorporation of GFP-hCAP-G into the condensin I complex was confirmed by co-IP with anti-GFP antibody and the precipitates were probed with a mixture of antibodies against hCAP-C, hCAP-E, hCAP-D2, and hCAP-H. The co-IP pattern was compared to that of the untransfected HeLa extracts using anti-hCAP-G antibody.

Figure S2

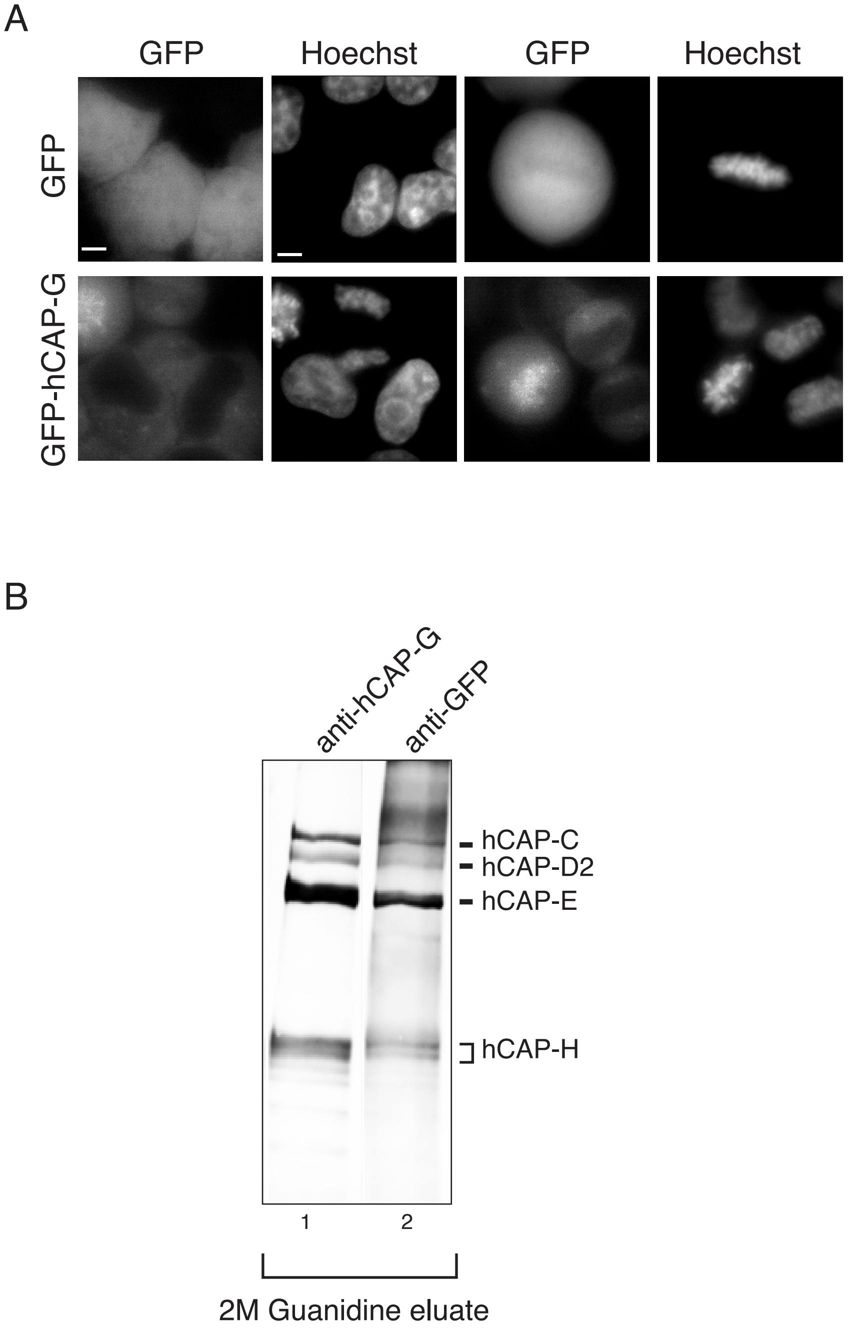
A
GFP
Hoechst
GFP
Hoechst
GFP
GFP-hCAP-G
B
anti-hCAP-G
anti-GFP
hCAP-C
hCAP-D2
hCAP-E
hCAP-H
1
2
2M Guanidine eluate
