# Supplementary material for: Condensin I Recruitment to Base Damage-Enriched DNA Lesions Is Modulated by PARP1
Source: PLoS One. 2011 Aug 12;6(8):e23548. doi: 10.1371/journal.pone.0023548 (PMC3155556; doi:10.1371/journal.pone.0023548)

Figure S3. Chromatin association of condensin I in PARP1 knockout cells. The wild type and PARP1 knockout mouse embryonic fibroblasts (MEFs) [1] were treated with or without H_2O_2 and chromatin fractions were purified by CSK extraction [2] and probed for mouse CAP-D2 and PARP1 by western blotting.

1. Wang ZQ, Auer B, Stingl L, Berghammer H, Haidacher D, et al. (1995) Mice lacking ADPRT and poly(ADP-ribosyl)ation develop normally but are susceptible to skin disease. Genes & Dev 9: 509-520.
2. Heale JT, Ball J, A. R., Schmiesing JA, Kim JS, Kong X, et al. (2006) Condensin I interacts with the PARP-1-XRCC1 complex and functions in DNA single-stranded break repair. Mol Cell 21: 837-848.

Figure S3

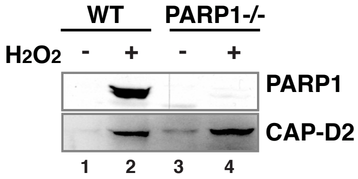

Supplement: Figure S3 — Chromatin association of condensin I in PARP1 knockout cells. The wild type and PARP1 knockout mouse embryonic fibroblasts (MEFs) [26] were treated with or without H2O2 and chromatin fractions were purified by CSK extraction [14] and probed for mouse CAP-D2 and PARP1 by western blotting. (PDF) [file pone.0023548.s003.pdf]
